# Contributions of Nitric Oxide to AHR-Ligand-Mediated Keratinocyte Differentiation

**DOI:** 10.3390/ijms21165680

**Published:** 2020-08-08

**Authors:** Carrie Hayes Sutter, Haley M. Rainwater, Thomas R. Sutter

**Affiliations:** 1 Department of Biological Sciences, University of Memphis, Memphis, TN 38152, USA; hmrnwter@memphis.edu (H.M.R.); tsutter@memphis.edu (T.R.S.); 2 W. Harry Feinstone Center for Genomic Research, University of Memphis, Memphis, TN 38152, USA; 3 Department of Chemistry, University of Memphis, Memphis, TN 38152, USA

**Keywords:** keratinocyte, differentiation, aryl hydrocarbon receptor (AHR), 2,3,7,8-tetrachlorodibenzo-*p*-dioxin (TCDD), nitric oxide synthase (NOS), nitric oxide (NO), *S*-nitrosylation, metabolic reprogramming, reactive oxygen species (ROS), reactive nitrogen species (RNS)

## Abstract

Activation of the aryl hydrocarbon receptor (AHR) in normal human epidermal keratinocytes (NHEKs) accelerates keratinocyte terminal differentiation through metabolic reprogramming and reactive oxygen species (ROS) production. Of the three NOS isoforms, NOS3 is significantly increased at both the RNA and protein levels by exposure to the very potent and selective ligand of the AHR, 2,3,7,8-tetrachlorodibenzo-*p*-dioxin (TCDD). Inhibition of NOS with the chemical *N*-nitro-l-arginine methyl ester (l-NAME) reversed TCDD-induced cornified envelope formation, an endpoint of terminal differentiation, as well as the expression of filaggrin (FLG), a marker of differentiation. Conversely, exposure to the NO-donor, *S*-nitroso-N-acetyl-DL-penicillamine (SNAP), increased the number of cornified envelopes above control levels and augmented the levels of cornified envelopes formed in response to TCDD treatment and increased the expression of FLG. This indicates that nitric oxide signaling can increase keratinocyte differentiation and that it is involved in the AHR-mediated acceleration of differentiation. As the nitrosylation of cysteines is a mechanism by which NO affects the structure and functions of proteins, the *S*-nitrosylation biotin switch technique was used to measure protein *S*-nitrosylation. Activation of the AHR increased the *S*-nitrosylation of two detected proteins of about 72 and 20 kD in size. These results provide new insights into the role of NO and protein nitrosylation in the process of epithelial cell differentiation, suggesting a role of NOS in metabolic reprogramming and the regulation of epithelial cell fate.

## 1. Introduction

Ligand activation of the aryl hydrocarbon receptor (AHR), a basic-helix-loop-helix, Per-Arnt- Sim domain-containing transcription factor, is associated with diverse cellular, immunological, and toxicological effects [1]. Particular to keratinocyte and skin models, activation of the AHR results in an accelerated differentiation program [2,3], as well as an increase in cellular proliferation [4]. In monolayer and organotypic cultures of keratinocytes, activation promotes the differentiation of the proliferating basal cell, ultimately leading to an increase in terminally differentiated anucleated corneocytes [5,6,7,8]. In utero, activation of the receptor accelerates the development of the murine epidermal permeability barrier [8,9]. 

Activation of the AHR increases the expression of numerous genes involved in keratinocyte differentiation, yet decreases the expression of an even larger number of genes, several of which have been linked to keratinocyte differentiation [5,7,8]. The repression of two of these genes, SLC2A1 and ENO1, results in a decrease in glycolytic flux and an increase in SIRT1, which are necessary for keratinocyte differentiation [10]. Similar to repression of genes in the glycolytic pathway, activation of the AHR decreases the expression of numerous mitochondrial genes. This repression likely contributes to the decreased inner mitochondrial membrane potential, decreased ATP production and increased mitochondrial-specific reactive oxygen species (ROS) in the differentiating keratinocyte. Importantly, inhibition of ROS with antioxidants blocks AHR-mediated keratinocyte differentiation [7]. Thus, as a regulator of glycolysis and mitochondrial function, the AHR metabolically reprograms the keratinocyte and alters its cell fate. This oxidative environment of the differentiating keratinocyte is conducive to the formation of a subset of ROS, reactive nitrogen species (RNS). 

Nitric oxide (NO) is produced by the conversion of L-arginine and oxygen into l-citrulline. This reaction is catalyzed by the three nitric oxide synthase enzymes, NOS1, NOS2, or NOS3. Each of these isoforms are homodimeric, calmodulin-binding oxidoreductases that couple with nicotinamide-adenine-dinucleotide phosphate (NADPH), heme, tetrahydrobiopterin (BH4), flavin adenine dinucleotide (FAD), and flavin mononucleotide (FMN) to facilitate the transfer of electrons between their oxygenase and reductase domains. In addition to substrate and cofactor availability, NOS enzymatic activity is controlled by transcriptional, post-transcriptional, translational, and post-translational regulation [11,12,13]. While NOS1 and NOS2 are broadly expressed across tissues, the expression of NOS3 is narrower. NOS1 and NOS3 are normally expressed constitutively, although they are also inducible. The expression of NOS2 is most often detected following exposure to certain inflammatory cytokines and/or bacterial polysaccharides, but it is also constitutively expressed [14]. In the skin, constitutive NOS activity is believed to play a role in the epidermal barrier as well as in the blood flow rate in the microvasculature, while induced NOS activity is important in wound repair, responses to UV irradiation, pathogens, and the etiology of certain skin diseases [15]. 

The biological signaling of NO has two main pathways. The first is the classical mode where NO binds to heme iron on soluble guanylate cyclase to induce the formation of cGMP. The induction of cGMP affects enzymes such as cGMP-gated ion channels, phosphodiesterases, and protein kinase G, resulting in vasodilation, immune responses, and other biological responses. The second pathway involves post-translational modification of proteins by nitrosylation. In the presence of ROS, NO forms RNS that mainly target cysteine, tyrosine, and tryptophan amino acid residues [13]. The *S*-nitrosylation reaction with cysteine is the most common as it has the fastest kinetics. It affects the structure and function of a diverse set of protein substrates [13]. *S*-nitrosylation is exceedingly recognized as an important posttranslational modification relevant to signal transduction and similar to phosphorylation in its specificity [16]. *S*-nitrosylation of certain mitochondrial proteins is known to affect mitochondrial function and increase ROS [17].

In the absence of growth factors, keratinocytes in culture differentiate as the level of confluence increases; calcium accelerates this differentiation [18]. AHR activation with 2,3,7,8-tetrachlorodibenzo-*p*-dioxin (TCDD) promotes these mechanisms of keratinocyte differentiation [5]. RNA microarray data from normal human epidermal keratinocytes (NHEKs) indicates that activation of the AHR increases the expression of the nitric oxide synthesizing enzyme, NOS3 [7], suggesting that NO and likely RNS are increased in the differentiating keratinocyte. Here we investigate these possibilities and the effect on keratinocyte differentiation.

## 2. Results

**Figure 1:**
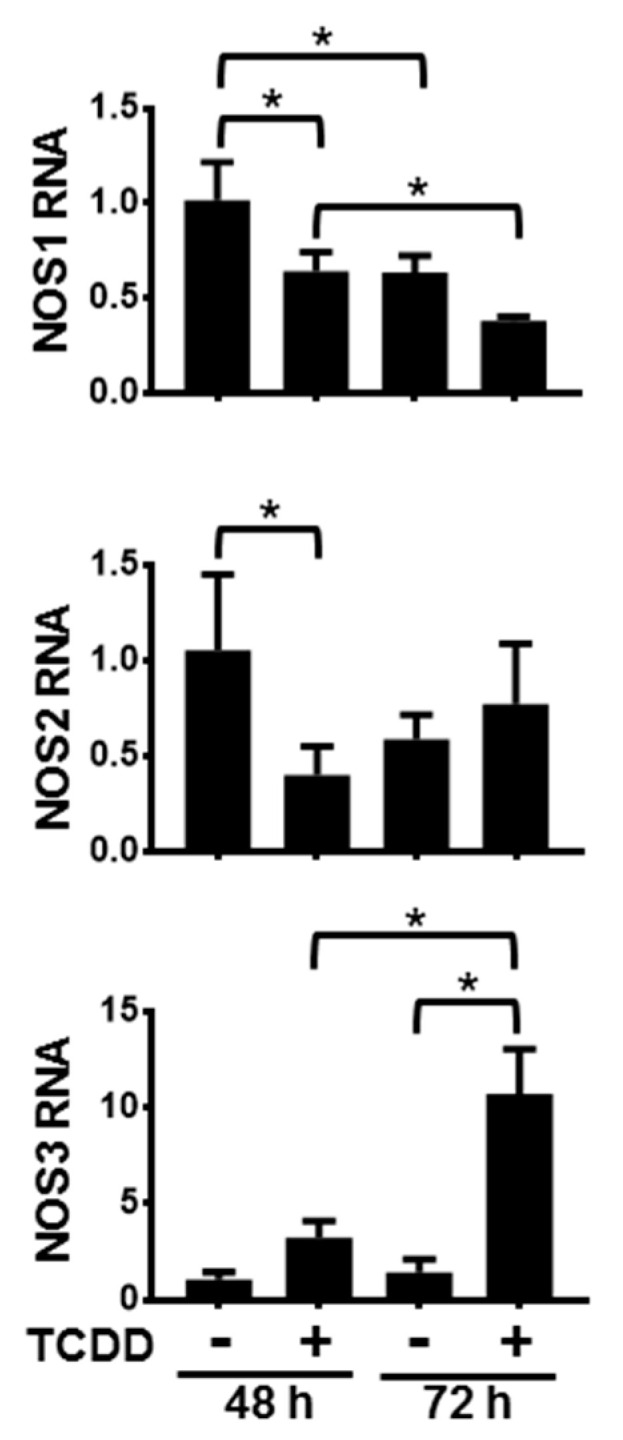
Activation of the aryl hydrocarbon receptor (AHR) alters the levels of NOS1, 2 and 3 RNA. mRNAs from normal human epidermal keratinocytes (NHEKs) treated with dimethyl sulfoxide (DMSO) (0.1%, vehicle) or TCDD (10 nM) for the indicated times were measured. Levels (mean (*n* = 4) +/- SD) were normalized with TATA binding protein (TBP) and plotted relative to DMSO (48 h), set to a value of one. * indicates a significant difference, *p* < 0.05. Data were analyzed using two-way ANOVA followed by Tukey’s post hoc tests.

Activation of the AHR in NHEKs had varying time-dependent effects on the levels of NOS mRNAs (Figure 1). Following 48 h and 72 h of treatment with TCDD, NOS1 levels significantly decreased compared to dimethyl sulfoxide (DMSO)-treated controls. Additionally, levels of NOS1 following 72 h of DMSO as well as 72 h of TCDD were significantly lower than their respective 48 h levels, indicating that NOS1 gene expression decreased during keratinocyte differentiation. Activation of the AHR accelerates this decrease. After 48 h of exposure to TCDD, NOS2 RNA levels were also decreased. By 72 h of TCDD treatment there was a slight, but not significant increase of NOS2 RNA levels. Additionally, NOS2 levels in the 72 h DMSO-treated samples were not significantly decreased compared to levels in the 48 h DMSO-treated samples, indicating that NOS2 RNA is not significantly changing during keratinocyte differentiation. In contrast, levels of NOS3 RNA increased approximately 3-fold over the levels of the time-matched DMSO control following 48 h of TCDD exposure, and approximately 7-fold over the levels of the time-matched DMSO control following 72 h of TCDD treatment. The control levels of NOS3 at 72 h increased slightly compared to 48 h control time point, though not significantly. These data point towards an increase of NOS3 RNA during keratinocyte differentiation in response to activation of the AHR. In summary, NOS1 RNA decreased during differentiation and this decrease was greater following activation of the AHR; NOS2 RNA transiently decreased in response to AHR activation; and NOS3 RNA increased during differentiation in response to activation of the AHR.

**Figure 2:**
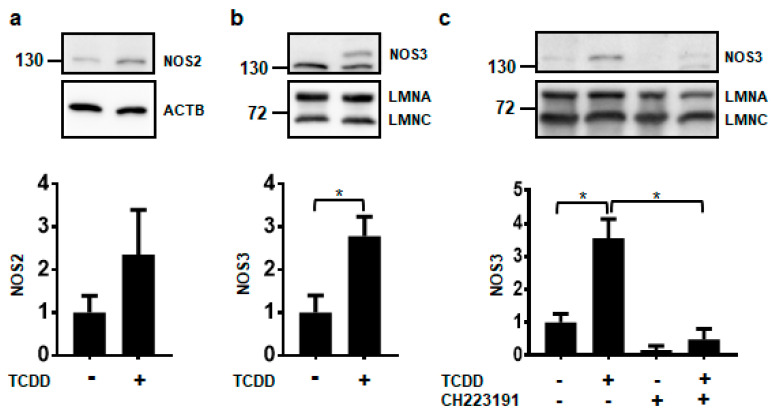
Activation of the AHR increases the expression of NOS3 protein. Protein levels were measured from NHEKs treated with DMSO (0.2%, vehicle) or TCDD (10 nM) for 72 h (**a**–**c**). In (**c**) NHEKs were also treated with the AHR antagonist, CH223191 (1 μM). Levels of NOS2 (**a**) and NOS3 (**b**,**c**) (mean (*n* = 3) +/- SD) were quantified by densitometry, normalized with levels of either Actin Beta (ACTB) or Lamin A (LMNA), as indicated, and plotted relative to DMSO, set to a value of one (lower panels). The numbers to the left correspond to molecular weight markers in kilodaltons. * indicates a significant difference, *p* < 0.05. Data were analyzed using two-tailed Student’s *t*-test (**a**,**b**) or two-way ANOVA followed by Tukey’s post hoc tests (**c**). These results were replicated in NHEKs from a different source.

Of the three NOS isoforms, only NOS2 and NOS3 proteins were detected in NHEKs using our experimental conditions (Figure 2). It is currently unknown whether the levels of NOS1 in NHEKs were below the limit of detection or whether the antibody did not have enough specificity and selectivity to detect NOS1 by immunoblot (data not shown). Further investigations of NOS1 will need to be pursued before a conclusion on its protein levels can be made. Although NOS2 levels appear to be increasing by TCDD, this increase was not statistically significant (Figure 2a). Only the NOS3 protein was significantly increased by ligand activation of the AHR about 3-fold, consistent with NOS3 RNA increases (Figure 2b). TCDD-mediated increases in the NOS3 protein were abrogated by cotreatment with the potent and specific antagonist of the AHR, CH223191 [19], providing additional evidence that the effect of TCDD is mediated through the AHR (Figure 2c). These data indicate that of the NOS enzymes, NOS3 may selectively contribute to keratinocyte differentiation. 

**Figure 3:**
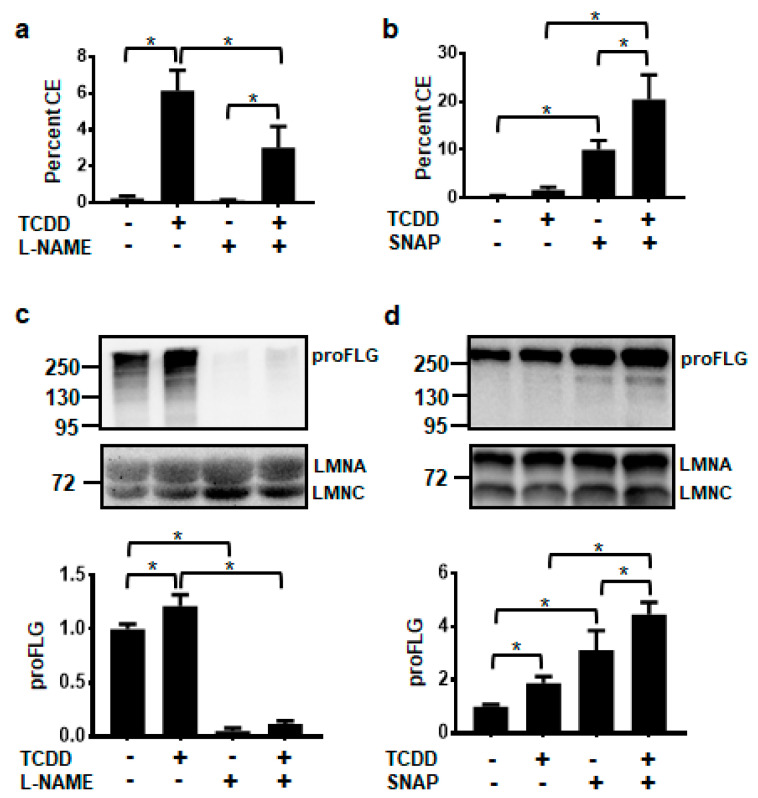
AHR-mediated keratinocyte differentiation is decreased by the NOS inhibitor, l-NAME (1 mM) (**a**,**c**) and increased by the NO donor, SNAP (100 μM) (**b**,**d**). Percent cornified envelope (CE) formation (**a**,**b**) (mean (*n* = 4) +/- SD) was measured in NHEKs treated as indicated for 5 days. Levels of unprocessed FLG protein, proFLG, (**c**,**d**) (mean (*n* = 3) +/- SD) were measured in NHEKs treated as indicated for 72 h. Protein was quantified by densitometry, normalized with levels of LMNA, and plotted relative to DMSO, set to a value of one (**c**,**d**, lower panels). The numbers to the left correspond to molecular weight markers in kilodaltons. * indicates a significant difference, *p* < 0.05. Data were analyzed using two-way ANOVA followed by Tukey’s post hoc tests. These results were replicated in NHEKs from a different source.

The non-isozyme specific NOS inhibitor, *N*-nitro-l-arginine methyl ester (L-NAME), and the NO donor, *S*-nitroso-N-acetyl-DL-penicillamine (SNAP), were used to determine whether levels of NO affected keratinocyte terminal differentiation, as measured by the cornified envelope (CE) assay. TCDD significantly increased cornified envelope formation to 6%. Treatment with L-NAME alone did not significantly alter the formation of CEs, but cotreatment of l-NAME plus TCDD decreased the percentage of CEs to one-half the level of TCDD alone (Figure 3a). Conversely, exposure to only the NO donor, SNAP, increased CE formation compared to the DMSO control and cotreatment of TCDD plus SNAP resulted in an even larger percentage of cornified envelopes (20%) (Figure 3b). Levels of the differentiation marker, filaggrin (FLG), correlated with cornified envelope formation. l-NAME decreased, while SNAP increased the levels of FLG in both the vehicle- and TCDD-treated NHEKs (Figure 3c,d).

**Figure 4:**
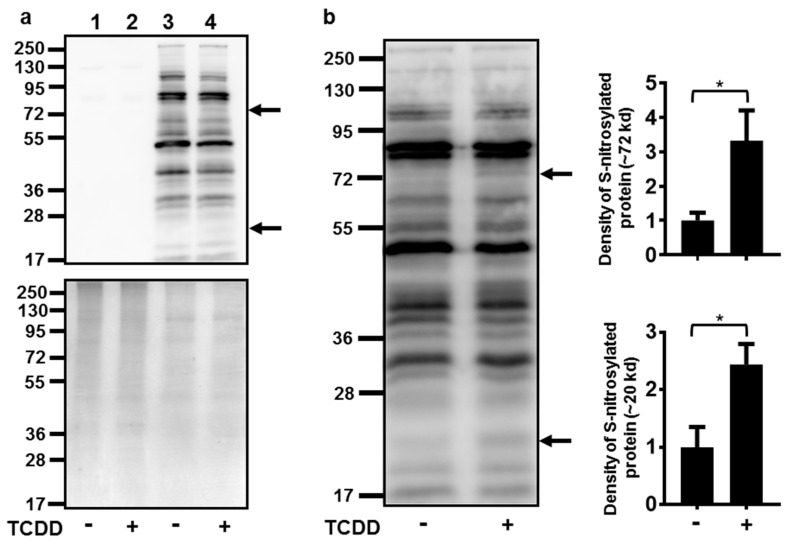
Activation of the AHR increased the *S*-nitrosylation of a 72 and a 20 kD protein. (**a**) Controls for *S*-nitrosylation biotin switch assay. Upper panel immunoblot of samples without performing the switch assay (lanes 1 and 2) to determine the assay background and with performing the switch assay (lanes 3 and 4). Treatment with TCDD (72 h, 10 nM) is as indicated. The arrows to the right of the immunoblot images indicate the proteins with increased *S*-nitrosylation. Numbers to the left correspond to molecular weight markers in kilodaltons. The upper band corresponds to a protein ~72 kD and the lower arrow, a protein ~20 kD. Lower panel, the same samples as in the upper panel were run in parallel and stained with coumassie blue to demonstrate similar protein loading of samples. (**b**) Quantitation of protein *S*-nitrosylation. Left, immunoblot of *S*-nitrosylated proteins. Treatment with TCDD is as indicated. The numbers to the left correspond to molecular weight markers in kilodaltons. The upper arrow corresponds to a protein ~72 kD and the lower arrow corresponds to a protein ~20 kD. Right, levels of protein *S*-nitrosylation of ~72 kD and ~20 kD proteins (mean (*n* = 4) +/- SD) were quantified by densitometry, and plotted relative to DMSO, set to a value of one. * indicates a significant difference, *p* < 0.05. Data were analyzed using two-tailed Student’s *t*-test.

Numerous attempts to indirectly measure NO production in NHEKs using the Greiss assay (with and without conversion of nitrate to nitrite) were unsuccessful, as the levels of nitrite in NHEKs were below the detection limits of these assays (data not shown). Due to this detection limitation and our knowledge of increasing ROS at the time of NOS3 induction [7], an assay to detect cellular targets of RNS, protein *S*-nitrosylation, was undertaken. Activation of the AHR increased the *S*-nitrosylation of two proteins with apparent molecular weights of approximately 72 and 20 kD (Figure 4). The *S*-nitrosylation of each of these proteins increased about 3-fold.

## 3. Discussion

Metabolic regulation of cell fate is described for several biological systems including macrophages [20], stem cells [21], vascular endothelial cells [22], and T cells [23]. Our recent studies [10] demonstrate that AHR-mediated keratinocyte differentiation occurs through a series of metabolic reprogramming events including a decrease in glycolysis by the down-regulation of the glucose transporter, SLC2A1, and the glycolytic enzyme, ENO1. The decrease in glycolytic flux stimulates an increase in SIRT1 that is reversed by pyruvate supplementation. Furthermore, AHR-mediated keratinocyte differentiation is dependent on this increase in SIRT1. Down-regulation of glycolysis using glycolysis pathway inhibitors has the similar effect as activation of the AHR on keratinocyte differentiation, demonstrating that a decrease in glucose metabolism is sufficient to affect keratinocyte cell fate. In addition to glycolysis, activation of the AHR affects mitochondrial oxidative phosphorylation causing ATP levels and inner mitochondrial membrane potential to decrease. Concomitantly, the ratio of GSH/GSSG decreases, consistent with lower levels of glutathione reductase activity, and mitochondrial ROS levels increase. Importantly, inhibition of ROS with antioxidants decreases the AHR-mediated acceleration of differentiation [7]. Consistently, epidermal deletion of the murine mitochondrial transcription factor A (TFAM) diminishes mitochondrial ROS and inhibits epidermal differentiation [24]. Due to the significant contribution of ROS to keratinocyte differentiation [7,24] and because RNS are critical effectors of oxidation-potential that often target mitochondrial proteins [17], an understanding of RNS in the regulation of the keratinocyte cell fate is of interest. 

Here we have followed up on the observation that AHR activation increases the levels of NOS3 RNA in keratinocytes [7]. To begin our understanding of a role of NO in AHR-mediated keratinocyte differentiation, all three NOS isoforms, NOS1, NOS2 and NOS3, were investigated. Of the three isoforms, only NOS3 RNA and protein were consistently and significantly increased by activation of the AHR in keratinocytes. AHR binding was not detected within 5 kb of the transcriptional start site of NOS3 [10], suggesting that the increase of NOS3 by the AHR is not due to direct transcriptional regulation, but to a factor capable of inducing NOS3. To the best of our knowledge, AHR-mediated effects on NOS3, specifically in keratinocytes, have not been reported. In murine fibroblasts, TCDD increases NOS3 RNA [25]. However, in primary human umbilical endothelial cells AHR agonists decrease NOS3 activity and NO formation, and NOS3 expression is increased in the aortas of AHR -/- mice compared to WT mice [26]. Thus, in certain cells and tissues, activation of the AHR appears to repress NOS3 expression, in contrast to what we report here in keratinocytes. In regard to the other NOS isoforms, activation of the AHR increases NOS1 in PC12 cells [27] and NOS2 in microglial [28], mesenchymal stem cells [29], and murine lung homogenate [30]. The mechanistic studies in the lung homogenates indicate that NOS2 is not a direct gene target of the AHR, as binding of the AHR to the NOS2 gene was not detected. The authors propose that a NOS2-inducing factor mediates the increases by TCDD [30]. 

Through the use of the non-isozyme specific NOS inhibitor, l-NAME, and the NO donor, SNAP, we provided evidence that NO plays a role in AHR-dependent and AHR-independent acceleration of terminal differentiation. Previous reports of the involvement of NO in keratinocyte differentiation are conflicting. For example, our results of the involvement of NO in keratinocyte differentiation are in sharp contrast to a study demonstrating an inhibition of CE formation following SNAP exposure [31]. This may be due to the differences in concentration of SNAP, as inhibition of CE formation was reported using concentrations of SNAP 10-100X higher than the 100 μM used here. This suggests that the effects of NO on keratinocyte differentiation may be concentration dependent. Additionally, differences may reflect peroxynitrite formation, which is dependent not only on NO, but also on specific ROS, such as superoxide. For example, the pure NO donor, 3-(2-Hydroxy-2-nitroso-1-propylhydrazino)-1-propanamine (PAPA NONOate), while inhibiting cell growth, does not increase keratinocyte differentiation markers to the same extent as the NO donor, 3-morpholinosydnonimine (SIN-1), which increases both NO and peroxynitrite. Consistently, peroxynitrite itself increases the markers of keratinocyte differentiation [32]. Therefore, a better understanding of the importance of each RNS and each ROS and their cellular concentrations is necessary for the interpretation of responses to NO.

Somewhat less controversial is the anti-proliferative effect of NO in keratinocyte models. An increase in NO formation either by LPS/IFNγ, TH-1 cytokines, or by the NO donor, SIN-1, decreases keratinocyte cell growth and several markers of proliferation [32,33,34]. Additionally, the strong proliferative factor, EGF, inhibits the formation of NO and NO-induced growth suppression [34]. These data indicate that the activities of EGF and NO oppose each other, and that EGF increases keratinocyte cell growth at least partially by repressing the formation of NO. This anti-proliferative response to NO may be concentration dependent as it was demonstrated that higher levels of NO decrease the proliferative marker, Ki67, while lower levels of NO increase it [35].

Here we demonstrate that activation of the AHR increases protein *S*-nitrosylation. Specifically, we observed an increase in the *S*-nitrosylation of a 72 and a 20 kD protein. Although the identification and importance of these proteins are still under investigation, an increase in the *S*-nitrosylation of these proteins indicates that RNS are increased during AHR-mediated keratinocyte differentiation, concomitant with an elevation of mitochondrial ROS [7]. *S*-nitrosylation of mitochondrial proteins increase ROS and cause respiratory changes [17] reminiscent of the effects of AHR activation. Future studies will focus on the *S*-nitrosylation of mitochondrial proteins and their possible involvement in the mechanism of AHR-mediated keratinocyte differentiation.

In summary, we demonstrate that AHR-mediated increases in NOS3 expression correlate with increases in protein *S*-nitrosylation. Thus, it appears that AHR-mediated increases in NOS3 are not merely increasing levels of NO, but also levels of RNS, which is consistent with the oxidizing environment of the differentiating keratinocyte. Moreover, we provide evidence that keratinocyte differentiation is dependent on NO. This suggests that in keratinocytes, reactive nitrogen species are a component of the reactive oxygen species required for AHR-mediated terminal differentiation. 

## 4. Materials and Methods 

### 4.1. Cell Culture

Neonatal NHEKs were purchased from Lonza (Walkersville, MD, USA). NHEKs were grown as previously described [5,36,37]. Cells were grown to confluence before pretreatment in basal keratinocyte serum free media (KSFM) (17005042, ThermoFisher, Hanover Park, IL, USA) for 24 h, followed by treatment in basal KSFM with 1.8 mM CaCl_2_ for the time and with chemicals indicated. 

### 4.2. Chemicals

The following chemicals were used DMSO (D2650, MilliporeSigma, Billerica, MA, USA), L-NAME (1 mM, 80210-1, Cayman Chemical, AnnArbor, MI, USA), SNAP (100 μM, 82250-10, Cayman Chemical, AnnArbor, MI, USA), CH223191 (1 μM, C8124, MilliporeSigma, Billerica, MA, USA) and TCDD (10 nM).

### 4.3. RNA Isolation and Quantitative PCR

For all RNA studies, cells were treated for 48 or 72 h, as indicated. Total RNA was isolated using RNA Stat-60 (Tel-Test, Inc., Friendswood, TX, USA). The cDNA was made using oligo dT primers (IDT, Coralville, IA, USA) and M-MLV Reverse Transcriptase (28025021, ThermoFisher, Hanover Park, IL, USA) following the manufacturer’s instructions. Quantitative PCR (qPCR) reactions were performed using iQ SYBR Green Supermix (28025021, Bio-Rad Laboratories, Hercules, CA, USA). Target RNA levels were normalized to values of TATA binding protein (TBP). The efficiency of each primer set was determined and used in the quantitation [38]. The melt curve analyses of the amplicons resulted in single peaks. The primers (IDT, Coralville, IA, USA) used in this study were the following: NOS1FP: CCCTCTCTTGGACTTCAGGG; NOS1RP: ATGGAAACAACCACTGGGCT; NOS2FP: CGCATGACCTTGGTGTTTGG; NOS2RP: CATAGACCTTGGGCTTGCCA; NOS3FP: CGAGTGAAGGCGACAATCCT; NOS3RP: CGAGGGACACCACGTCATAC; TBPFP: ATGCCCTCCTGTAAGTGCCC; TBPRP: TAGCAGCACGGTATGAGCAA. 

### 4.4. Immunoblotting, Enhanced Chemiluminescence (ECL), and Densitometry

Following treatment, cells were collected in ice-cold PBS and pellets were lysed in ice-cold whole cell lysis buffer (0.1% SDS, 1% NP40, 5 mM EDTA, 0.5% sodium deoxycholate, 150 mM NaCl, 50 mM Tris-HCl pH 8) supplemented with phenylmethylsulfonyl fluoride and protease inhibitor cocktail (P8340, MilliporeSigma, Billerica, MA, USA). The samples were spun for 10 min at 10,000× *g* at 4 °C and the supernatant was used for immunoblotting of NOS2 and NOS3, ACTB, and LMNA/C. To measure FLG, cell pellets were boiled for 5 min in lysis buffer (62.5 mM Tris-HCl pH 6.8, 2% SDS, 1% 2-beta-mercaptoethanol). The samples were spun for 10 min at 10,000× *g* at room temperature and supernatant was used to detect FLG. Protein was quantitated using the Pierce Micro BCA kit (23235, ThermoFisher, Hanover Park, IL, USA). Proteins were separated by PAGE (7% gel, 150 Volts) and transferred to polyvinylidene fluoride membranes (30 Volts, overnight, 4 °C). Blocking and antibody incubations were in Tris-Buffered Saline (pH 7.6) with 0.1% Tween 20 containing 5% nonfat dry milk. Following incubation with Clarity Western ECL Substrate (1705061, Bio-Rad Laboratories, Hercules, CA, USA) bands were visualized using the ChemiDoc Touch Imaging System (Bio-Rad Laboratories, Hercules, CA, USA). Signal density was quantitated using Image Lab software (v5.2.1) (Bio-Rad Laboratories, Hercules, CA, USA). 

### 4.5. Antibodies

Primary antibodies used for immunoblotting: NOS2 (NB300-605, 1:1000, Novus, Littletown, CO, USA); NOS3 (ab5589, 1:1000, Abcam, Cambridge, MA, USA); FLG (NBP1-87527, 1:2000, Novus, Littletown, CO, USA and PRB-417P-100 1:1000, Covance, Princeton, NJ, USA); LAMIN A/C (4777, 1:5000, Cell Signaling Technology, Danvers, MA, USA), ACTB (A5441, 1:100,000, MilliporeSigma, Billerica, MA, USA). The HRP-conjugated secondary antibodies were used for immunoblotting: goat anti-rabbit (111035144, 1:10,000, Jackson ImmunoLaboratories Research, West Grove, PA, USA) and goat anti-mouse (115035003, 1:10,000, Jackson ImmunoLaboratories Research, West Grove, PA, USA). 

### 4.6. CE Assay

Confluent NHEKs were exposed to SNAP (100 μM), l-NAME (1 mM), TCDD (10 nM), or a combination of the chemicals as indicated for 5 days in basal KSFM plus 1.8 mM CaCl_2_. Additional SNAP was added to the media every 24 h, and additional L-NAME was added at 48 and 72 h. The formation of CEs, a measure of terminal differentiation, was quantified essentially as previously described [39]. NHEKs were counted before they were spun and resuspended in solution containing 10 mM Tris-HCl pH 7.5, 1% SDS and 1% 2-mercaptoethanol. CEs were counted following a 10-min incubation at 90 °C. The number of CEs were determined per number of cells and expressed as a percentage. 

### 4.7. S-Nitrosylation Assay

*S*-Nitrosylation of proteins was measured following the manufacturer’s instructions for the Biotin Switch Assay (*S*-nitrosylation) (ab236207, Abcam, Cambridge, MA, USA) [40]. Proteins (2.5 μg) were separated by PAGE (10% gel, 150 Volts) and transferred to polyvinylidene fluoride membranes (30 Volts, overnight, 4 °C). Blocking and reagent incubations were in Tris-Buffered Saline (pH 7.6) with 2% BSA. Following incubation with Clarity Western ECL Substrate (1705061, Bio-Rad Laboratories, Hercules, CA, USA) bands were visualized using the ChemiDoc Touch Imaging System and the signal density was quantitated using Image Lab software (v5.2.1, Bio-Rad Laboratories, Hercules, CA, USA). 

### 4.8. Statistical Analysis

Statistical analysis was performed using Prism 7.03 (GraphPad, San Diego, CA, USA). The use of each test is described in the legends of each figure.

## Abbreviations

AHR	Aryl hydrocarbon receptor
CE	Cornified envelope
ECL	Enhanced chemiluminescence
DMSO	Dimethyl sulfoxide
KSFM	Keratinocyte serum free media
L-NAME	N-nitroarginine methyl ester
NHEK	Normal human epidermal keratinocyte
NO	Nitric oxide
RNS	Reactive nitrogen species
ROS	Reactive oxygen species
SIN-1	3-morpholinosydnonimine
SNAP	*S*-nitroso-N-acetyl-DL-penicillamine
TCDD	2,3,7,8-tetrachlorodibenzo-*p*-dioxin
